# New Energy Geographies: A Case Study of Yoga, Meditation and Healthfulness

**DOI:** 10.1007/s10912-014-9315-3

**Published:** 2014-12-13

**Authors:** Chris Philo, Louisa Cadman, Jennifer Lea

**Affiliations:** 1 School of Geographical and Earth Sciences, University of Glasgow, Glasgow, UK; 2 Faculty of Development and Society, Sheffield Hallam University, South Yorkshire, UK; 3 School of Geography, University of Exeter, Exeter, UK

**Keywords:** Energy/energies, Energy geography/geographies, Yoga/meditation, Affect/affective energies, Health and healthfulness

## Abstract

Beginning with a routine day in the life of a practitioner of yoga and meditation and emphasising the importance of nurturing, maintaining and preventing the dissipation of diverse ‘energies’, this paper explores the possibilities for geographical health studies which take seriously ‘new energy geographies’. It is explained how this account is derived from in-depth fieldwork tracing how practitioners of yoga and meditation find times and spaces for these practices, often in the face of busy urban lifestyles. Attention is paid to the ‘energy talk’ featuring heavily in how practitioners describe the benefits that they perceive themselves to derive from these practices, and to claims made about ‘energies’ generated during the time-spaces of these practices which seemingly flow, usually with positive effects, into other domains of their lives. The paper then discusses the implications of this energy talk in the context of: (a) critically reviewing conventional approaches to studying ‘energy geographies’; (b) identifying an alertness to the likes of ‘affective energies’ surfacing in recent theoretically-attuned works of human geography (and cognate disciplines); and (c) exploring differing understandings of energy/energies extant in geographical studies of health and in step with the empirical research materials presented about yoga, meditation and healthfulness. While orientated towards explicitly geographical inquiries, the paper is intended as a statement of interest to the wider medical humanities.

**Her day**

**07.00**: She wakes up in her flat and does a handful of yoga sun salutations. She feels that this practice enlivens her, helping her to face the day, making her feel ‘energetic’:^1^

… it is a good way of checking in every day, how am I today, how am I feeling? And then you can actually tailor your days to how you’re feeling: **are you feeling very energetic**, are you feeling down, are you feeling tired? So, you can think, instead of just getting up and going out, … I’m feeling this today, so I might be careful of doing that; or I might think, oh, I’ve got **lots of energy** today, I can do lots of things today. (Diarist 19)

She also knows how differently she feels on mornings without yoga practice:^2^

Felt quite tired this morning. I can really tell the difference on the days I don’t practice i.e. more tired, sluggish, **less energy**, and it makes me thankful and more appreciative that I do have a regular practice to sustain me and give me that **energy boost** in the morning. (Diarist 1 entry, 26/05/10)

**08.00**: She walks to work, briskly through the park, meditating a little under the trees. She calls it her ‘meditative walk’, creating good ‘mental energy’:

… the benefits [are] not just physical …, but also helping with general mental health … a **positive energy level**. … On a physical level, cardiovascular, … [and] **the mental aspect of extensive enlivened energy** as well as a sense of calm, and when I can do the meditation it also helps centre oneself. It’s just that **energy level** with that physical benefit of the yoga practice. (Diarist 26)

**09.00**: She starts her day’s work at the office, entailing lots of meetings, some stressful. She senses her ‘energy levels’ to be high, partly thanks to the preceding moments of yoga/meditation,^3^ allowing her to deal effectively with work tasks:

Well, being in the business …, it’s important for me to be **full of energy** … to keep one’s **energy level** high … [W]hen one’s motivating oneself, … to … get out of bed in the morning after 3 hours sleep or get through a long day of being [at work], **keeping the energy levels up**; and I realised quite quickly that doing things like Pranayama didn’t just have physical benefits, it also had mental benefits like concentration and **energy levels**, which I found extremely valuable. (Diarist 26)

She feels that she is *using* her energy in a good way, using ‘*good*’ not ‘*bad* energy’ and avoiding:

… kind of **using your energy wrong**, … you know, a lot of my life I've just been running from one thing to another like in a frantic kind of excited way because I’m [a] quite excitable person, **energetic sort of person**, frenetic, I would say, and gregarious, you know. (Diarist 25)

**13.00**: She takes a lunch-hour, leaving her work premises to sit quietly alone on a seafront wall, meditating while gazing out to sea. It creates in her a feeling of calm relaxation, ‘emptying out’ the ‘mental clutter and ‘centering the self’ in the middle of a high-pressured day:

… emptying out when you’re having a busy day or you’re faced with a busy day, it’s just emptying out as well as centred. So the mind is … obtaining relaxation … . In the … hustle and bustle of a modern western life, it is actually [more] difficult to relax through a daily routine than it otherwise would be in a more peaceful lifestyle. So relaxation … **mental energy** and … a state of being centred, not self-centred … with a capital ‘S’, as opposed to me, me, me, me, I want me, me, me. (Diarist 26)

**17.00**: She attends her bi-weekly yoga class at the nearby Natural Health Centre. A number of other folk arrive, and there is a ‘buzz’ or ‘energy’ about the session:

So I think it’s very important to have that space to go …, because **the energy in a room** makes such a difference, you know. Just the fact that there’s all those other people and that you’ve all come there together, and you are all there for … similar reasons. And there’s just **an amazing energy in a room** and particularly in the self-practice class because all you can hear is just people breathing. And everyone is just totally focussed on what they are doing and, yeah, **it**’**s just energising** and uplifting; just being there makes a difference. (Diarist 1)

**18.00**: She lingers a while after class, chatting with an instructor and then having coffee with other participants. She learns more about the ‘energies’ of the mind-body, as well as continuing to feel the ‘energy’ of an enthused community:

… he was talking about the **energy body**, **feel the energy**, **feel the energy** in your body over and over again … (Diarist 3)

Coffee afterwards at Brighton Coffee Company, Kensington Gardens – ‘There was **a lovely energy afterwards** and lots of adrenaline flowing.’ (Diarist 1 entry, 23/05/11)

**19.00**: She gets the bus home, feeling tired but relaxed, physically and mentally. Despite being a little sleepy, she still feels ‘energised’. An hour later, she arrives home, cooks dinner with her partner, and is about to slump in front of the TV but instead decides to tackle household chores. She is mildly surprised that she has the ‘energy’ to do them:

At home, reading and doing household chores: ‘Feeling rested and **energetic**.’ (Diarist 2 entry: 25/05/10)

Cooked / watered plants: ‘No tiredness, aches or pains! Feel **energetic** and able to accomplish tasks.’ (Diarist 2 entry: 15/06/10)

**23.00**: She goes to bed, reflecting on a ‘fine day’ when everything seemed to go well. She gently drifts off to sleep full of a sense of healthy well-being.

**A project**

‘Her day’ here is a compendium of events, experiences and sensations described by participants in a research project conducted by the authors.^4^ Based in Brighton and Hove (henceforth just ‘Brighton’), a south coast UK city inhabited by many practitioners of so-called ‘new spiritual practices’ of the self,^5^ our project explores how people may negotiate such practices in their daily lives (Heelas 2008; also Heelas et al. 2005). This case study approach allows investigation of these spiritual practices as ones embedded within, rather than separate from, the context of ongoing everyday lives, routines and spaces.^6^ We consider how yogic and meditative practices are ‘fitted into’ daily schedules (how successfully and sustainably) and how they possibly ‘leak out’ to shape other facets of a person’s life and work (positively or even negatively). Running throughout is attention to whether our practitioners conceive of their practices as enhancing personal senses of healthy well-being, physically, mentally and (if harder to specify) spiritually. Furthermore, the horizon for our inquiry is a standard conceptualisation of increasingly busy, segmented and stressful ‘modern’ lives, particularly as lived in large urban areas whose very ‘ecology’ – the distribution of activities, splitting asunder of home and work, and continual nagging demands, embodied and en-minded—is constantly chipping away at times and spaces which might be dedicated to ‘care of the self’.

In-depth interviews were conducted with teachers (n = 23) and pupils (n = 14) of yoga and meditation, and the audio-tapes were transcribed and then coded according to various emergent themes (‘energy’ being but one). Practitioners of yoga and meditation (n = 26) were recruited to keep a detailed diary on the days when they practised, providing data on the form, duration and location of their practice and also invited to reflect on how these practices articulate with their (often very busy) urban routines.^7^ These diaries were processed to disclose how the daily time-paths traced by participants through the city produce a diversity of effects (perhaps in bodily relaxation but also maybe aches and pains) and affects (emotionally-charged ‘effects’ permeating body-psyche continuums). On the one hand, we seek to recreate the ‘time-geographies’ (Pred 1977) of daily movement and encounter; and on the other, we respond to recent trajectories within (and beyond) human geography that stress the ‘phenomenology’ of how bodies sense and are ‘affected’ by their corporeal dwelling within the world (eg. Lea 2006, 2008, 2009, 2011; Simonsen 2013).^8^

The diary methodology has influenced the opening section of our paper, which uses the chronology of a day to introduce the theme of ‘energies’: of everyday worlds, tracked through mundane time-spaces, traversed by what our participants themselves describe as the creation, maintenance, heightening and dipping of ‘energy’ (levels). Virtually all of the transcripts and diaries are punctuated by ‘energy’-type references, and the fractured effect at the outset of our paper—broken up with indented quotations and quote-marked terms or phrases – is designed precisely to *show* something of our primary data (and to dispel any suspicion that we are imposing an ‘energy’-laden interpretative grid on to our participants). That said, ‘her day’ here is indeed a composite representation, gathering together quotes and extracts from a number of different participants’ interviews and diaries, and thus there is a measure of artifice in depicting ‘her day’ as so thoroughly imbued with flows of (and occasional blockages to) energies seemingly released by new-spiritual practices. Indeed, ‘her day’ here is *fuller* of explicitly yogic/meditative events and energies than was the case for most participants, although, for a few, their whole days *were* not far off being this fully framed through the lens of such practices.^9^ Caveats aside, therefore, we reckon that, for most of our participants, their days really are living experiments in ‘energy geographies’, albeit they might not all explicitly cast what they are doing as wilful experimentation.^10^

## Talking and time-spacing energy

As underlined above, the vocabulary used by our participants in interview and when writing diary entries is replete with terms like ‘energy’ and other words possessing a close family resemblance (feeling ‘lively’, ‘revitalisation’ and ‘rejuvenation’). We hear repeatedly about individuals wanting to boost their ‘energy levels’, to become ‘energised’, to be ‘energetic’; about their senses of good (positive) energy and bad (negative) energy, or at least about mobilising energies in good or bad ways; about their feeling the ‘energy’ of being with others, enjoying the same kind of events; about energies that are both (perhaps simultaneously) physical and mental; about, more mysteriously, feeding upon the ‘energies’ that certain practices appear to unlock within the body-psyche; or, even, tapping into ‘energies’ somehow outwith the body-psyche, floating in the atmosphere, in ‘nature’, all around us.

There are clearly *diverse* meanings of energy being deployed here, and a key finding is that our participants are thoroughly *eclectic*, we might say *hybrid*, in the knowledges which they are conjoining (from moment to moment, in a single sentence, but also across a life-course). Sometimes participants are using the term energy in the utterly familiar sense of feeling that one’s physical body is full of energy, able to do lots of stuff, with plenty of stamina, likely *not* experiencing aches and pains. Conversely, there are times when they talk of being bereft of energy, in short being exhausted, perhaps carrying aches, pains and injuries. Our diaries contain numerous moans about *over*-doing that yoga session, pushing too far on that pose, and hence depleting one’s energy stocks. The picture is thus not always rosy.

As should be obvious from our brief listing above of energy talk, there are also many instances when participants depart from what might be taken as a conventional – we might say biomedical – take on energy. Thus, we find ourselves hearing about energies as an *alternative* account of bodily and mental well-being largely alien to Western biomedicine. As Jennifer Lea explains in her paper on massage:

While energy is an inexact and non-unified way of describing and understanding the body, the basis of the idea is of a flow around, through and beyond the body. This gives rise to a body that is understood as essentially unbounded by the skin and is rather open to flow and transfer with other objects, people and environments. The understanding is that when this flow is blocked, illness and suffering can arise. It is these blockages that massage works on to re-establish free flow and restore health. (2011, 5)

For the more strictly ‘scientific’ persona,^11^ such talk may appear loose, sloppy even, perhaps metaphysical. For such a persona, moreover, the claim would probably be that we should regard these vocabularies as largely *metaphorical*, since understandings of energy as the animating force of *physical systems* are simply being colonised to describe what is occurring in the hybrid corporeal, psychological, emotional and spiritual ‘system’ that is the human being. Indeed, such a persona might speculate that, once science arrives at a full biomedical explanation (and ‘mapping’) of the body, then it will be possible to give a properly ‘scientific’ reasoning for at least some of the more ‘spiritual’ beliefs about body-psyche energies held by Lea’s massage practitioners or participants in our Brighton study. Such contrasting understandings of energy/energies will resurface later in our discussion.

Let us briefly return to ‘her day’ and to the window opened on how our participants – *not* merely as ‘individuals’ but also as ‘collectivities’ mutually influencing each other’s life-worlds—are consistently ‘energising’ themselves around a variety of *sites* where yoga/meditative practices are occurring. Such sites may be formally set aside for such practices: ‘classes’ or ‘sessions’ in a natural health centre, a Buddhist centre, a church hall or even a municipal leisure centre. Other sites might be found ‘at home’, when waking up or going to sleep, or perhaps when more deliberately reorganising a home-space (clearing the children’s toys, for instance, to lay down the yoga mat or placing a Buddha on the mantelpiece). Other sites again might be ones with a connection to outdoor landscapes – parks, rivers, the seaside—with the notion of ‘life-energies’ flowing from and across said spaces into and through human bodies (as one meditates on a bench or while walking, cycling or swimming, or even as one practices a few yoga moves on a sunny park-slope). A further dimension is how individuals carve dedicated times-spaces from the press of often fraught urban daily/weekly routines for conducting their yogic/meditative practice: in which regard participants underscored the need to create instances of ‘stillness’, apart from the normal pacing of thought-and-activity, when energies could indeed be recuperated – in effect, created anew – as resources to be deployed in the wider struggle to cope, to accomplish healthfulness.^12^

Here, indeed, we find what we describe as the everyday ‘energy geographies’ of our participants, ones which – while presented highly empirically at the start of our paper – nonetheless resonate remarkably with a variety of more conceptually-driven notions now entering the broader fields of both human geography and, if as yet to a lesser extent, medical humanities (eg. Aho 2008; Gibson 2006; Rossiter 2012). Perhaps in partial reversal of normal academic procedure, it is arguably less that we possessed a prior theoretical agenda for probing the qualitative data generated by our research and more that this data almost ‘invites’ us to consider particular theoretical possibilities for bringing into dialogue with our Brighton study. Indeed, even more than this, materials from our research arguably help us better to understand certain realms of theory rather than vice versa, which arrive ‘after the event’ of the research. It is in this slightly unusual vein that we now make what we fully acknowledge as an abrupt shift in register, since we now wish to leave the empirics behind and spend the remainder of the paper travelling a route from the ‘energy geographies’ depicted above into a more conceptually-facing discussion of a minor ‘energy turn’ arguably now occurring within human geography, geographical health studies and, potentially, the wider medical humanities.

## Discussion: towards animate ‘other energies’

### Old energy geography

There is a long-standing if now less often explicitly named subfield known as *the geography of energy* or *energy geography*. Its focus has been the spatial distribution of energy sources and supply—the spaces of energy production and consumption; the locational dynamics of the energy sector/industry—delineating global and regional patterns in, and ‘landscape features’ resulting from, the exploitation of fossil fuel deposits, natural gas reserves and hydro-electric power with some nods to alternative energy sources (solar, wind, wave and biofuels). In Anglophone geography, John D. Chapman wrote in 1961 about ‘a geography of energy: an emerging field of study;’ Gerald Manners authored in 1964 the first book-length treatment entitled *The Geography of Energy*; and F.J. Calzonetti and B.D. Solomon edited in 1985 a volume entitled *Geographical Dimensions of Energy*, describing it as the “first attempt to provide a comprehensive volume on the topic of energy geography” (ix).

Explorations of energy geography had already occurred in France, notably by Pierre George with his 1950 scholarly work *Géographie de l*’*énergie*, but also in texts by the perhaps more well-known Max Sorre. We stress this fragment of historiography because the Anglophone authors, unlike their French predecessors, have been clear that they are concerned with so-called *inanimate energy* (eg. Chapman 1961, 10). Indeed, Manners (1964, 21) explicitly narrowed his focus to inanimate energy, acknowledging that “[i]n the writing of the French geographers in particular (George 1950; Sorre 1948, 209 ff.) discussion of energy has tended to include some references to the use of *animate energy* [our emphasis].” The distinction between these two forms of energy is clarified here:

For our purposes, energy will be disaggregated into two sub-categories, namely E_a_(t), animate (ie. muscular) energy, and E_i_(t), inanimate energy … . Examples of the animate energy include human and animal force (ie. muscular force), while examples of inanimate energy include internal combustion, steam power, wind power, hydraulic power and electrical power. (Beaudreau 1998, 42)

This sharp distinction between studying inanimate and animate energy has indeed mapped through into Anglophone geography, setting up a sole tendency to contemplate the energy dimensions of humans and other life-forms in terms of their position within *ecosystems* responding ultimately to the harnessing and transformation of ‘radiant energy’ from the sun. Anthony Hoare defined energy as “the ability to do work,” adding that it “can be channelled into four forms, three of them (thermal, mechanical and electrical) end-forms and one (fuels) a holding operation for future work” (1979, 507). The forms of energy gathered into the bodies of human beings themselves, as they convert nutrition into ‘muscular force’, are hence accorded scant attention. Despite significant shifts in how scholars now characterise the subfield of energy geographies (eg. Bridge 2012; Zimmerer 2012),^13^ as linked to radical rethinking of ‘resource geographies’ (eg. Bakker and Bridge 2006), the centralising of the human as itself a locus and source of animate energy (or, indeed, of other life-forms in this respect) has not been considered.

### New energy geographies

At the same time, though, quite other conceptions of energy – we might say of ‘other energies’ – have started to appear in the orbit of contemporary human geography and cognate disciplines often with links across to realms of health and medicine. A range of manoeuvres under the umbrellas of post-phenomenological, non-representational, vitalist and emotional geographies have all carried an under-current of concern for ‘energies’, never that explicitly stated and somewhat buried in (often densely-written) paragraphs, yet arguably amounting to an intimation of ‘new energy geographies’. Set within a lineage leading from Spinoza and the Romantics to a raft of Deleuzian-inspired writings, the notation of *affect* – as already name checked – has been central to this development. To caricature swathes of debate, affect is here identified as a sensation which ‘moves’ between peoples and perhaps other life-forms, a flow of possibilities for feeling ‘something’ differently which circulates in the ‘atmosphere’ of a given place, preceding its localisation in an individual’s emotional register (where it might also become available for reflection and ‘wording’) but possessing a definite generative potential (with the capacity to prompt *trans*-individual effects). Seen as such, affect has the capacity for producing changes in individual and even collective human (and maybe non-human) conduct, and it is therefore unsurprising that the vocabularies of ‘energy’, usually pluralised as energ*ies*, are occasionally enlisted to assist in the visualisation of what is occurring.

Political philosopher William Connolly, whose account of ‘neuropolitics’ (2002) is a common reference-point for discussion of affective geographies:

Affect consists of relatively mobile energies with powers that flow into conscious, cultural feeling and emotion; yet these affective energies also exceed the formulations they help to foment. Affect has an element of wildness in it. … Emotion with no affect would be dead, merely a pile of words as empty containers; emotion and mood filled with affect brim over with energy-potentials that exceed ready-made articulations. (Connolly 2011, 151)

The ‘energy talk’ here is conspicuous, and it resonates with passages from Nigel Thrift, a key geographer of affect, when speaking of how affect can “allow the release of positive energies” (2004, 76) or when wondering about “viral models of contagion … posited as explaining the workings of a range of phenomena, … but often in highly speculative ways that posit a kind of performative energetics without specifying what the source, content or form of that energy might be” (nd, np). The speculative, non-specific character of such models, echoed it might be said in Thrift’s own texts, is what would bother more orthodox students of energy, of course, but it has not prevented fragments of such energy talk appearing elsewhere. Michele Lobo (2013) proposes to be “thinking of ethical engagement in terms of the ‘wildness’ of affective energies on the beach;” Gerda Roelvink (2010, 117) borrows from Connolly (2002, 76) to describe how testimonies at a 2005 World Social Forum session “triggered moments of ‘affective energy’ for creative thinking;” Jamie Lorimer (2012, 599) briefly alludes to “the affective energies of conservationists;” and Clive Barnett (2012, 380), if in a critical mode, explores how “[e]thics emerges from the ‘Continental’ tradition as a residue of hope, as a vitalistic energy to be enacted differently (perhaps in ways which cannot be anticipated).” Once such work is laid alongside strands of post-phenomenological geography, as evidenced in Lea’s research (Lea 2008, 2009, 2011) on bodies in yogic and massage spaces, the case for a proliferation of energy talk is strengthened. Indeed, while rarely expressed in quite this manner, ‘energies’ are actually central to Lea’s interest in how environments, places and spaces of all kind insinuate their own ‘ecologies’, entailing multiple assemblages of the human and non-human, as affective influences entering muscle and psyche, sinew and soul. In sum, then, it might even be plausible to speak of a minor ‘energy turn’ in contemporary human geography (which, ironically, has virtually nothing to do with a subfield called ‘energy geography’).

### Re-energised health geographies?

Insofar that scholarship in health geographies – meaning geographical inquiries into health-related subject-matters – has engaged at all systematically with energy matters, it has done so through a biomedical understanding of human energy. For instance, a quick survey of the journal *Health and Place* (founded in 1995) reveals fifty or more papers tackling the phenomenon of ‘obesogenic environments’, with a common emphasis (eg. Harten et al. 2008) on neighbourhoods – notably urban spaces with few green spaces, walkways, cycle lanes and the like, often in conjunction with clusterings of ‘fast food’ outlets and ‘cheap food’ stores – which are hostile to local inhabitants exercising, being energetic and hence healthfully expending energy (to avoid weight-gain). The focus effectively becomes a geography of embodied ‘physical’ energy levels, linking into deeper if often unspoken biomedical assumptions about what makes for spatial variations in healthy and unhealthy human metabolisms (and here a connection can be spied with Sorres’s constructs of ‘biological geography’ and the ‘pathogenic complex’: see Akhtar 2003).

What our survey of *Health and Place* also reveals, however, is a handful of papers where ‘energy’ is deployed to index more emotionally-, psychologically- and even spiritually-charged modalities of energy that also display geographical associations. In his piece on Lourdes, Wil Gesler (1996, 103) discusses a woman pilgrim, wholly depleted of energy by a demanding home-life, who departed the site with her energy levels (physical and spiritual) recharged; and then in his piece on ‘words in wards’, the same author discusses the “positive healing energy” released for a particular suburban New Jersey community by “use of the name of Jesus, saying mantras or uttering the phrase ‘*I am*’” (1999, 19). A study into the ‘ecologies’ of a rural retreat concludes that “[m]any of the interviewees found that spending their time at Holton Lee lifted their mood, whil[e] also raising energy levels before return to their home-setting” (Conradson 2005, 345); a study into ‘taskspaces’ at a camp for troubled youth found participants talking about “[t]he group’s energy [being] low” or reflecting on realisations of how to “use a lot of my negative energy to make something, you know, good” (Dunkley 2009, 94); and a study considering school ‘arts and health’ experiments, heard a teacher bemoaning “[v]ery jumpy energy – half-term coming up” (Atkinson and Robson 2012, 5). Other authors relate the case of *onsen*, hot springs in Japan fed by geothermal energy (a possible theme for conventional energy geography), whose visitation for slow, prolonged immersion “was a cherished activity a few decades ago. Farmers used to take yearly breaks and to visit hot springs, which recharged them with energy for the next seasons” (Serbulea and Payyappallimana 2012, 6).

The energy talk here is not foregrounded in these papers, it is true, but arguably comprises a rumbling sub-text of encountering ‘other energies’ which, if only in the thought-worlds of particular peoples and places, seemingly hold implications for health and well-being. More sustained in its attention to such energies is Ronan Foley’s (2011) inspired inquiry into Irish holy wells as ‘therapeutic assemblages’ comprising diverse spatial concatenations of people, things, symbols and behaviours. As he remarks:

More usefully, energies, a term hinted at but under-used .., is a key ingredient in this spatial stew, especially when associated with health and healing. Material settings and bodies hold within them a range of potential energies, those energies have symbolic healing associations and it is through energetic performances of health in place that productive aspects of the assemblage … are expressed. (2011, 472)

The waters in the wells might really be quite ‘ordinary’, but they are embedded within a matrix of “deeper meanings, many of which were associated with the energies of the place itself and its affective reputation as a site of renewed energies” (475). Such claims can perhaps re-energise, as it were, how the likes of Gesler addressed the ‘therapeutic landscapes’ of Lourdes and other healing sites, but at the same time they explicitly chime across to the more theoretically-driven notations of affective energies, after Connolly, Thrift and others, as outlined previously. They announce the possibility for new energy geographies permeating geographical studies of health whose guiding compass is less (Western) biomedicine and more all of the ‘other energies’ which run through ecologies, bodies and minds affording (or occluding) qualities and practices of health, healing and well-being.

These would be alternative health studies for whom animate energy is central; studies prepared to risk taking seriously yogic and meditative spaces (to give just one example) as the gathering and distribution points for ‘other energies’ (holistically, so it seems, operating on both the [deceptive] materiality of bodies and the [apparent] immateriality of minds).^14^ There are indeed exciting new energy geographies to ponder, some but not all with definite ramifications, many with a health-therapeutic dimension, all as disclosed by our own research and introduced (more-or-less explicitly) in the empirical sections above. As possibilities to consider: *chakric geographies*, concerned about an awareness of chakras as spiritual-physical energy centres and lines, energetic regions and routes, across, through and even maybe reaching beyond the human body; *geographies of environmental connectedness*, a key thread in Lea’s recovery of Scaravelli’s yogic teachings about how “the function of the Earth is to collect energy from the ground” (2008, 94); *geographies of mental energy*, released by ‘mindful’ meditational attentiveness to the countless micro-spaces of body, self and world, particularly if facilitated by the creation of stillness, slowness and focus (*not* multi-focus, multi-tasking); ‘*atmospheric*’ *geographies*, in part after David Conradson (2003, 2005, 2011), where energies seemingly fizz and settle in ‘a room’ of intense yogic or meditative practice, maybe lingering a while afterwards; or even *gentle geographies* of lightness, humility and care, maybe as a re-approximation of the anarchist energies of the ‘gentle geographer-prince’ (Kropotkin).

These are strange notations perhaps – ones ideally needing elaboration; ones readily open to a more structural critique of inequalities damaging people’s well-being, for which our musings about new energy geographies could seem irrelevant – but we hope it can be agreed that our reflections, firmly rooted in a sustained burst of empirics centring on what research participants reveal about the *energies* energising *their* own life-worlds, do at least speak four-square to a bigger theme, surely central to an enlarged medical humanities, of diverse energies and prospects for cultural explorations of health and well-being. If medical humanities has long excelled in unpacking the textual play of terms like ‘energy’ in the words (written but sometimes spoken) of medics and patients, taking seriously how “we think in metaphors and act accordingly” (Shoeneman et al. 2012, 187), then there are clear potentials for interfacing a medical humanities sensibilities with the energy talk of our project participants (but possibly also of the scholars cited above). If medical humanities is also open to considering alternative perspectives on the multiple materialities of the world *beyond* its textual inscriptions, perhaps contemplating what it means to speak after Deleuze about ‘force’ as “a fundamental flow of energy” (Gibson 2006, 185), then there are other potentials for bringing medical humanities and ‘new energy geographies’ – and perhaps the likes of ‘her story’ from the start of this paper – into energetic conversation.

## Endnotes

^1^ Unattributed in-text phrases (between single quote marks) are ones typically deployed by many of our interviewees and diarists. Indented quotations derive either from our diarist-interviewees or from entries written in their own diaries (with dates of an entry given). Emboldened words or phrases indicate our emphases.

^2^ Extracts in Arial typeface derive from diaries kept by research participants; bold text indicates our emphasis. We asked our diarists to keep a diary on days when they practised yoga (or meditation), the idea being that they reflected on how the practice maybe influenced other aspects and parts of their day. Occasionally, diarists reflected on what it meant not to practice on a given day or according to their normal routine for such practice.

^3^ While a contemporary, and arguably Western, distinction can be made between modern postural yoga (hitherto ‘yoga’) and modern meditational yoga (hitherto ‘meditation’), for the purpose of the present paper we have chosen to unite the two, largely because the ‘energy talk’ here traversed both terrains of practice. For a discussion on differences between modern postural yoga and modern meditational yoga, see De Michelis (2005) and Connolly (2007).

^4^ Details about project, including working/draft papers, are available at: http://spiritualitiesresearch.wordpress.com/ (also Cadman et al. 2014).

^5^ We are aware that there are various social axes – of class, gender, ethnicity, sexuality, (dis)ability –fracturing any singular experience of yoga/meditation, and our research attends closely to such fractures in our empirical database. They are not pulled to the fore here, albeit we should acknowledge the preponderance of women over men diarists/interviewees. Despite the opening section being headed ‘Her day’, though, some of the quotes/extracts derive from male participants.

^6^ There is a deeper critical-theoretical logic to our inquiry, in that we are seeking to insert matters of *geography* into research on contemporary spiritualities, insisting on sustained alertness to regional *milieux* within which such beliefs and associated practices are formed and enacted – in cryptic summary, taking seriously that Brighton UK is *not*, say, Blackburn UK – but also delving into the most intimate time-space geographies of how spiritual practices become woven into the everyday fabric of being, doing, moving and encountering.

^7^ We then recruited 14 of these diarists to do the in-depth interviews, which explicitly followed-up on what had been disclosed in their diaries: this was a deliberate two-stage methodology. For more on our project methodology, see Cadman et al. (2014).

^8^ There is a tangled realm of theoretical labour underlying these deceptively simple remarks, coupled to intense debates about: the ‘scales’ at which such claims can be advanced (including the implied scale-jumps from bodies to cities and back again); the processes whereby ‘affects’ become registered emotionally, and thereby available to be ‘worded’ by individuals, and differences in how such processes might be conceived (eg. phenomenologically as opposed to psychoanalytically); and, more broadly, the whole problematic of how materiality (as in tangible time-paths through sites and situations) articulates with immateriality (including domains of spirituality, affect and emotion). For clues about this complexity, see deliberations over ‘non-representational geographies’ in Anderson and Harrison (2010).

^9^ The ‘representativeness’ of our participants in relation to the wider population of Brighton (or the UK) cannot be understood in the classic mode of statistically-inclined social science; rather, this case, and the overall package of cases presented in our project, have to be appreciated as representing *nothing* (or at least *little*) other than itself. Instead, these cases are each ‘mini-laboratories’ for exploring connections and possibilities, a conclusion that stands even when scaling up to the collectivity of cases and, moreover, to talking about Brighton as a ‘new-spiritual city’.

^10^ For some, though, there is a clear sense of ‘experimenting’ with different possibilities for improving well-being, gaining spiritual insight or simply coping better with the daily round. Many had self-consciously been switching between different kinds of ‘new spiritual practice’ (and, indeed, also embracing ‘old’ religious worships as well as a diversity of psychotherapeutic, sporting and other possible activities) in pursuit of the ‘mix’ that best suited their individual needs and capacities (see also Lea et al. 2014).

^11^ We deliberately deploy this term, ‘persona’, to evoke, as one referee puts it, “the inevitably embodied nature of any view, rather than disembodying them (a ‘scientific perspective’)” (Referee #1).

^12^ A cynical view might be that here the activities of individuals are actually entirely conformable with the demands of modern ‘fast’ capitalism: “Practices of yoga/meditation are arguably easily fitted within the dominant temporal-spatial organisation of the body under capitalism (eg. the demand that we ‘fit in’ ‘care of the self’ in snippets of time), such that a certain underlying ethos of ‘productivity’ (of ‘maintaining’ or ‘boosting’ energy) is reinforced through practices, such as yoga, that might initially be seen as counter to the energy-depletions of capitalist work” (Referee #2). Our own sense is that, for our participants at least, a framing of such activities as dissident to such capitalist demands, and the usual spatial-temporal frames imposed on everyday lives, would be equally plausible. Such matters will repay further consideration in later publications.

^13^ Note the recent establishment of an Energy Geographies Working Group of the Royal Geographical Society with the Institute of British Geographers (see http://energygeographiesworkinggroup.wordpress.com/about/).

^14^ And also hinting at a meeting of West and East, always alert to a post-colonial critique of Western priorities and appropriations: a set of themes never far from the surface in our own research project.
